# Validation of indicators for the welfare assessment of captive Yangtze finless porpoises (*Neophocaena asiaeorientalis asiaeorientalis*)

**DOI:** 10.1017/awf.2025.19

**Published:** 2025-05-21

**Authors:** Sara Platto, Agathe Serres, Simona C. Normando, Xavier Manteca, Deborah Temple, Yujiang Hao

**Affiliations:** 1 Department of Biotechnology, College of Life Sciences, Jianghan University, Wuhan, PR China; 2 Sanya Key Laboratory of Marine Mammal and Marine Bioacoustics, Institute of Deep-sea Science and Engineering, Chinese Academy of Sciences, Sanya, PR China; 3 Department of Comparative Biomedicine and Food Science, University of Padua, 35020 Padua, Italy; 4 Department of Animal and Food Science, College of Veterinary Sciences, Universtat Autonoma de Barcelona, Barcelona, Spain; 5 AWEC, Edifici Eureka, Campus de la Universitat Autònoma de Barcelona, Barcelona, Spain; 6 Institute of Hydrobiology, Chinese Academy of Sciences, Wuhan, Hubei, PR China

**Keywords:** animal welfare, Five Domains model, mental state, welfare assessment protocol, Yangtze finless porpoises

## Abstract

The Five Domains model (FDM) is a widely accepted framework for developing welfare assessment tools across various contexts, including wild animals under human care. However, only two protocols have been developed for captive cetaceans. This study aimed to create a welfare assessment protocol based on the FDM for captive Yangtze finless porpoises (YFPs; *Neophocaena asiaeorientalis asiaeorientalis*). Indicators relevant to YFPs’ welfare were selected via a literature review, and validated through two consecutive questionnaire surveys, four discussions with a panel of three experts, and a blind review conducted by three additional cetacean welfare experts. This process resulted in the validation of 46 welfare indicators, which were used to develop the Yangtze Finless Porpoise-Welfare Assessment Protocol (YFP-WAP) which, in its final version, contains 150 indicators. Moreover, intensity levels (the degree of impact of each indicator on the porpoises’ welfare), valence (whether the indicator contributed positively or negatively to the porpoises’ welfare state), and mental states associated with each indicator were also assigned by the panels of experts. Additionally, a confidence score was assigned to each indicator’s intensity level, valence, and mental state that reflected experts’ level of uncertainty regarding the indicator impact on the YFPs’ welfare. This rigorous validation process provided transparency, helped ensure minimal bias, and reduced the likelihood of incorrect indicator elimination due to expert subjectivity. By integrating expert knowledge, the YFP-WAP provides a comprehensive approach to evaluating both positive and negative welfare states, supporting the ongoing care and management of YFPs in captivity.

## Introduction

Identifying the key elements of well-being from the animal’s perspective has long been a challenge, driving the development of diverse welfare assessment tools across species (Barnard & Hurst 1996; Hosey 2005; Dawkins 2006; Whitham & Wielebnowski 2013). These tools have played a crucial role in detecting potential welfare compromise, and guiding management practices to address species-specific issues (Webster 2005; Dawkins 2006; Blokhuis *et al.* 2010). While many welfare protocols are focused primarily on domestic animals, growing attention is being paid to the welfare of wild animals under human care, such as those in zoos and aquaria (Clegg *et al.* 2015; Mellor & Beausoleil 2015; Wolfensohn *et al.* 2015, 2018; Mellor 2017; Brando & Buchanan-Smith 2018; Fischer *et al.* 2021; Jones *et al.* 2022; Chavarría et al. 2023; Ghimire *et al.* 2024). Assessing welfare in these settings is complex and challenging due to the diverse biological and ecological needs of different species (Hill & Broom 2009; Kagan *et al.* 2015). Ensuring the welfare of wild animals in captivity is not only an ethical obligation but also supports the conservation, education, and research goals of the institutions housing them (Captive Breeding Specialist Group International Union for Conservation of Nature and Natural Resources, & International Union of Directors of Zoological Gardens 1993; Justice *et al.* 2017; Powell & Watters 2017; Brouwers & Duchateau 2021). Over the years, several welfare frameworks have been developed. Early models, based on the Five Freedoms, focused on improving nutrition, enclosure design, and health management (Hill & Broom 2009; Kagan *et al.* 2015). Some frameworks assessed welfare through direct assessment from keepers, while others evaluated the environment and management within facilities (Whitham & Wielebnowski 2013; Kagan *et al.* 2015).

Recently, the use of the Five Domains model (FDM) as a framework to build protocols for animal welfare assessment has increased (Mellor *et al.* 2020). This framework includes four physical specific domains: nutrition, physical environment, health, and behavioural interactions, which collectively influence the fifth domain — mental state (Justice *et al.* 2017; Sherwen *et al.* 2018). The FDM has gained popularity due to its versatility and applicability across different animal species and contexts (e.g. captivity or wild settings; Beausoleil *et al.* 2016; Mellor 2017; Harvey *et al.* 2020). The framework acknowledges the interaction between physiological and affective states in determining animal welfare (Mellor & Reid 1994; Hemsworth *et al.* 2011; Mellor 2016, 2019; Mellor *et al.* 2020). Although mental experiences are subjective and cannot be directly measured, they can be inferred from ‘welfare status’ indicators, which are animal-based measures (e.g. body condition, behaviours), and could offer evidence of what the individual may experience (Mellor & Reid 1994; Mellor *et al.* 2009; Mellor 2015a,b,c, 2017; Mellor & Beausoleil 2015; Hampton *et al.* 2016; Harvey *et al.* 2020; Serres *et al.* 2024). Inferences about animals’ mental states are supported by well-established knowledge from physiology, neuroethology, and affective neuroscience (Mellor & Beausoleil 2015; Mellor 2017; Harvey *et al.* 2020; Mellor *et al.* 2020; Hampton *et al.* 2023). On the other hand, ‘welfare alerting’ indicators, which can be either animal- or resource-based indicators (e.g. food availability, environmental conditions), reflect potential welfare risks within each domain (Harvey *et al.* 2020). Protocols based on the FDM do not aim to provide a precise welfare assessment for an individual animal, but to highlight critical elements that must be addressed for species-specific welfare improvements (Barber 2009; Ward *et al.* 2018). The model has been widely adopted for captive wildlife, including zoo animals (Kagan *et al.* 2015; Sherwen *et al.* 2018; Wolfensohn *et al.* 2018; Fischer *et al.* 2021; Jones *et al.* 2022; Chavarría *et al.* 2023; Ghimire *et al.* 2024), with recommendations from the World Association of Zoos and Aquaria (WAZA) (Mellor *et al.* 2015b).

Regarding captive cetaceans, a variety of anthropogenic (e.g. transport, noise, social isolation) and social factors (e.g. interactions with conspecifics: Serres *et al.* 2020b) may impact their welfare. Therefore, it is crucial to create tools to identify and address areas where welfare may be compromised or enhanced. While studies on cetacean welfare have focused on identifying possible behavioural (Clegg *et al.* 2015, 2017; Serres *et al.* 2020a; Miller *et al.* 2021a; Huettner *et al.* 2021), acoustic (Castellote & Fossa 2006; Stevens *et al.* 2021; Wong *et al.* 2023), cognitive (Clegg & Delfour 2018; Delfour *et al.* 2020; Ubeda *et al.* 2021), interaction with the trainers (Serres *et al.* 2022; Platto & Serres 2023), and physiological indicators (Pedernera-Romano *et al.* 2006; Serres *et al.* 2020a; Wong *et al.* 2023), very few comprehensive welfare assessment tools have been developed. To our knowledge, only the C-Well protocol, based on Welfare Quality® (Clegg *et al.* 2015), and the Dolphin-Wet protocol, based on the FDM (Baumgartner *et al.* 2024), have been developed which focus on bottlenose dolphins (*Tursiops truncatus*). Conversely, welfare assessment tools for other cetacean species, such as the critically endangered Yangtze finless porpoise (YFP; *Neophocaena asiaeorientalis asiaeorientalis*) are lacking.

The YFP, a subspecies of the narrow-ridged finless porpoise (*Neophocaena asiaeorientalis*), is endemic to the Yangtze River and adjacent lakes (Gao & Zhou 1993). After the presumed extinction of the Baiji (*Lipotes vexillifer*), the YFP became the only freshwater cetacean in the Yangtze River (Turvey *et al.* 2007). Following the sharp decline of the riverine cetacean population, from 2,500 in the early 1990s to just over 1,000 today, *ex situ* breeding programmes were established to help restore wild populations (Mei *et al.* 2014). A captive breeding programme was established in 1996 with the Chinese Academy of Sciences, the Yangtze Cetacean Breeding and Research Centre (YCBRC), which currently keeps twelve individuals under human care. In addition, other finless porpoise subspecies including the Indo-Pacific finless porpoise (*Neophocaena phocaenoides*), and the East Asian finless porpoise (*Neophocaena asiaeorientalis sunameri*) are kept in captivity in many aquaria in China and Japan. Given the critical need to ensure successful captive breeding and prevent extinction, developing tools to assess YFP welfare is essential. Understanding how individual animals respond to environmental, physiological, or husbandry factors can improve management practices, enhancing reproduction and overall well-being. The current project aimed to develop a welfare monitoring protocol for captive YFPs based on the FDM (Mellor *et al.* 2020), incorporating behavioural and physiological indicators from previous studies on the species (e.g. Serres *et al.* 2019, 2020a,b, 2022). The FDM was chosen as a guidance for its adaptability to various situations and species, and its proven effectiveness in identifying areas needing prompt interventions to improve animal welfare (Baumgartner *et al.* 2024).

## Materials and methods

**Figure 1.**
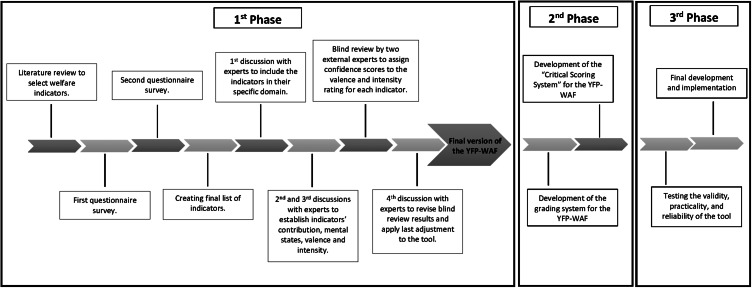
Diagram representing the different phases of the Yangtze Finless Porpoise-Welfare Assessment Protocol (YFP-WAP) development.

This manuscript is part of a wider study on the development of a Yangtze finless porpoise welfare assessment tool which includes three phases. The first phase – which is described in the current paper – includes five steps: (1) a literature review of potential welfare indicators for the considered species; (2) two questionnaire surveys; (3) four panel discussions with experts; (4) a blind review; and (5) the development of the structure of the welfare assessment tool (Jones *et al.* 2022; Serres *et al.* 2024). The second phase includes: (1) the development of a scoring system; and (2) the development of a ‘Critical Scoring’ pre-assessment check list which will be presented in a second manuscript. The third phase of the research project includes: (1) the evaluation of the validity, practicality, and reliability of the tool; and (2) the final development and implementation of the framework (Jones *et al.* 2022; Serres *et al.* 2024) (Figure 1). The primary objective of this protocol, based on the FDM, is to facilitate a structured, systematic, and comprehensive assessment of animal welfare, emphasising both welfare enhancement and comprise categories which highlight specific areas critical to the welfare of the YFPs (Mellor & Beausoleil 2015; Mellor 2017).

### Literature review and selection of the potential welfare indicators

The first step for the development of the YFP-WAP was to define an initial list of potential welfare indicators through the review of relevant literature. The welfare indicators were selected from previous studies on YFPs (Serres *et al.* 2019, 2020b, 2022) and the available literature on other cetacean species under human care (Clegg *et al.* 2015, 2018; Delfour *et al.* 2020; Baumgartner *et al.* 2024). This approach aligns with the ‘welfare analogy’ concept, which posits that knowledge of welfare in one species can inform understanding of the welfare of a related species with similar physiological and psychological functions, that have evolved and adapted to similar ecological pressures (Sandøe & Simonsen 1992; Sherwin 2001). The literature review was conducted through scientific literature databases (PubMed, Google Scholar), with no date of restriction, and selecting all relevant studies, written in English, and carried out on cetacean species with a focus on dolphin species under human care, where a combination of animal- and resource-based indicators were taken into consideration (Clegg & Butterworth 2017; Hampton *et al.* 2022; Beausoleil *et al.* 2023). Keywords such as “animal welfare assessment in dolphins” OR “welfare indicators in cetaceans in captivity” OR “cetacean welfare” OR “animal-based measures in dolphins” OR “dolphin welfare protocol” OR “welfare status indicators” OR “welfare alerting indicators” were used for the literature search. In general, an animal welfare protocol should include both animal-based measures that provide evidence of what an animal might experience, and indicators that provide information about possible future welfare risks (they can be either animal- or resource-based measures) in order to obtain a feasible and holistic welfare assessment, and to infer the animal’s likely mental/affective states (Rushen *et al.* 2011; Whitham & Wielebnowski 2013; Beausoleil *et al.* 2023). Moreover, when selecting welfare indicators, it is important to consider their feasibility, since the welfare assessment protocol might be used by personnel of the aquarium who are not researchers but mainly trainers and may be constrained by time or financial aspects (Mellor *et al.* 2020). Therefore, both animal- and resource-based measures were included and the practicality of indicators (i.e. can the indicator be reliably measured in captive YFPs groups) was assessed by SP, and AS who are familiar with YFPs, and the overall cetacean captive conditions.

A total of 37 welfare indicators were identified through the literature search and included in the initial protocol, covering a broad spectrum of animal- and resource-based measures.

### Expert opinion surveys

When the available literature on a species is limited, experts’ opinion represents a valuable method for identifying and validating indicators for animal welfare assessment (Rioja-Lang et al. 2020). In order to minimise the biases that come with the use of expert opinion, two consecutive rounds of questionnaire surveys were conducted. Panel members were selected based on their expertise in animal welfare science or their knowledge of dolphin welfare, behaviour, health, conservation, and YFP husbandry, as evidenced by their peer-reviewed publications (Hampton *et al.* 2023).

The 37 selected welfare indicators were used to draft the questionnaire for the first round of survey (Panel 1) (Ethical Approval: Ethical Approval: JXDXLL2024-083). The objective of the first survey was to ask the panel of experts to refine the indicator list by selecting which indicators to keep or remove, ensuring that only the most relevant measures for assessing YFP welfare were included in the final protocol. The survey was conducted online by using QuestionPro online software (Survey Software 2023). The questionnaire was structured in three parts:

1. In the first part of the questionnaire, experts were required to provide information regarding their education and experience, including one multiple choice question related to the expert field of work. In addition, three questions were used to assess experts’ knowledge about YFPs (“*How much knowledge do you have about Yangtze finless porpoises [YFPs]?”*), cetaceans in general (“*How familiar are you with cetacean species?”)*, and animal welfare (“*How familiar are you with the concept of animal welfare?”*) based on a five-point Likert scale;
2. In the second part of the questionnaire, experts were asked to respond with ‘Yes’, ‘No’, or ‘Unsure’ to the question: “*Should the following parameter be included as a welfare indicator?*” for each of the 37 potential welfare indicators. For each potential indicator, definitions were provided to ensure experts had the necessary information;
3. In the third part of the questionnaire an “*additional expert suggestions*” section was included to allow experts to provide comments or recommend indicators they considered important for the welfare assessment tool that were not present in the existing list.

The questionnaire link was sent by email to 57 experts to be completed anonymously within a one-month window. Experts could withdraw their consent at any time of the survey. A total of 30 completed questionnaires were collected, and indicators that received 60% or more of ‘Yes’ responses to the question “*Should the following parameter be included as a welfare indicator?*” were included in the framework. Twelve additional indicators suggested by the panel of experts were selected and incorporated into the revised questionnaire, bringing the total to 49. The new questionnaire, which followed the same structure as the previous one, was uploaded again on the QuestionPro online platform, and the survey link shared via email with 59 experts, including the 30 experts who participated to the first round (Panel 2). The aim of this second survey was to decide which indicators should be kept, and which additional indicators should be added to the list. The survey remained open for one month, during which 33 completed responses were collected and analysed. Indicators that received 60% or more ‘Yes’ responses to the question “*Should the following parameter be included as a welfare indicator?*” were included in the protocol. Moreover, 18 additional indicators were suggested by the experts to be included in the protocol. The list of indicators obtained from the second round of the survey were further evaluated over four discussions, and one blind review to create the definitive list used to build the YFP-WAP.

### Development of the structure of the YFP-WAP

A total of four separate group discussions involving the first two co-authors (one biologist expert in cetacean welfare and behaviour, and experienced on YFPs [AS], and a veterinarian expert in animal behaviour and welfare [SN]) and led by SP (veterinarian expert in cetacean welfare and behaviour, and experienced on YFPs) were conducted online. The discussion protocol was conducted according to Serres *et al* (2024). SP organised power-point slides, that were sent to AS and SN beforehand, that provided the basic information regarding the upcoming discussion, and required the participants to answer to some questions related to the topic under discussion. This protocol allowed the gathering of experts’ opinions and ideas prior to the upcoming discussion without them influencing each other. Participants had to return the completed document to SP prior to the meeting. The information gathered was used to frame the discussion session. The discussions always took place following the frame established by SP and ended once consensus was achieved. A blind review was conducted prior to the fourth discussion, including the opinions of three cetacean welfare experts who were not involved in the current study, and did not participate in the questionnaire surveys or discussions. The results from the blind review were then assessed during the fourth discussion.

#### First group discussion

During the first group discussion, experts were asked to determine whether to retain or discard the 18 additional indicators suggested by Panel 2 during the second questionnaire survey. Once the final list of indicators was established, the experts discussed which aspects of YFP welfare each indicator affected, and thus the domain(s) to which they should be assigned. Additionally, the panel discussed whether an indicator should be categorised as welfare status (WS) or welfare alerting (WA) based on the specific terminology of the FDM, and the domain to which it was assigned (Harvey *et al.* 2020). In the current protocol, some indicators could provide information on multiple welfare dimensions, and were therefore assigned to more than one domain. Depending on the indicator and its effect on a domain, it could be categorised as WS on one domain and WA on another. For example, “*general health*” was categorised as WS in domain 3 – Health as it directly reflects the individual health state, and as WA in domain 1 – Nutrition, and domain 4 – Behavioural Interactions since health can impact the YFPs’ appetite and social interactions. Consequently, these measures could have greater impact on the overall welfare assessment than measures linked to only one domain (Hörning 2001). This approach was preferred, because assigning each indicator to a single domain could introduce biases, limiting its interpretation to one specific type of affect rather than considering the broader context. Moreover, the redundancy that may arise from assigning an indicator to several domains is mitigated by interpreting its welfare implications distinctly for each domain (Bracke *et al.* 2002). In addition, experts also had to infer and attribute potential mental states to each of the selected welfare indicators. Potential mental states were inferred by experts using their knowledge about cetacean physiology, behaviour, health, and nutrition under human care.

#### Second and third group discussion

During the second and third discussions, experts assigned an intensity level to each indicator using a 3-point Likert scale: Low (1), Mild (2), and High (3). These intensity levels were determined based on the likely severity of the indicator’s impact, and the duration of its effect on the individual’s welfare. Intensity levels were assigned to each indicator, and they could vary depending on the domain to which the indicator was assigned. For example, the “*human-made noise disturbances*” indicator was attributed a Mild intensity level on domain 1 (Nutrition), Low on domain 3 (Health), and High on domain 4 (Behavioural Interactions). The experts also assigned a valence to each indicator, reflecting its positive or negative impact on YFP welfare. Precisely, based on the FDM specific terminology, an indicator could be categorised as welfare enhancement (WE) (+) or welfare compromise (WC) (–) (Harvey *et al.* 2020). Moreover, a confidence score was also assigned to each inferred mental state based on the evidence available in the literature, and on the knowledge of the experts. This confidence score reflects experts’ uncertainty about the validity of the inferred mental state, and ranges between 0 (no animal data available), 1 (low confidence), 2 (moderate confidence), and 3 (high confidence) (Beausoleil *et al.* 2016; Harvey *et al.* 2020; Baker *et al.* 2022).

#### Blind review and fourth group discussion

A blind review was conducted by three independent experts in cetacean welfare who were not involved in the questionnaire surveys or discussions. Experts were required to assign confidence scores to the valence and intensity level of each indicator based on the available literature, their knowledge, and experience. They were also asked to review the intensity levels and valence for each mental state, with the option to modify it if deemed necessary. The blind review process was used to mitigate bias due to the high level of familiarity SP and the two co-authors (AS and SN) had with the project. Following the review, a fourth discussion was organised by SP and AS and SN to evaluate the confidence scores assigned by the experts, and consider any last adjustments before finalising the protocol.

## Results

### Expert opinion surveys

In the first round of survey, experts (Panel 1) holding a PhD and possessing animal behaviour and animal welfare expertise represented the majority of the respondents. In addition, 86.7% of the experts were between somewhat familiar and extremely familiar with animal welfare, while 90% of the respondents were between somewhat familiar and extremely familiar with cetacean. Of the respondents, 63.3% had between fair and excellent knowledge of YFPs (for detailed information, see Supplementary materials S-1). In comparison to Panel 1, more experts involved in the second round of survey (Panel 2) held a PhD, while slightly more respondents had expertise on animal welfare. Compared to Panel 1, all respondents in Panel 2 had a certain familiarity either with animal welfare or with cetaceans, while 57.6% of the respondents had between fair and excellent knowledge about YFPs (for detailed information, see Supplementary material; S-1).

The first round of the survey allowed us to collect the opinion of 30 experts on the 37 indicators that were previously selected through literature review. No indicators were excluded during the first survey, and Panel 1 suggested adding 12 more to the initial list, which lead to a total of 49 welfare indicators included in the second round of survey. During the second round of the survey, out of the 49 indicators, 48 obtained at least 60% of ‘Yes’ responses to the question “*Should the following parameter be included as a welfare indicator?*” Only one indicator, “*training session duration*”, received less than 60% of ‘Yes’ responses, and was therefore removed from the list (Supplementary material; S-2). Moreover, Panel 2 recommended adding 18 new indicators to the 48 previously selected, resulting in a total of 66 indicators.

### Expert discussions

#### Selection and categorisation of indicators

The 66 indicators obtained from the second round of the survey were reviewed during the first discussion to ensure the absence of redundancy. The panel of experts decided to remove “*sex*” and “*age*” from the list of indicators, as they were not directly linked to any specific mental state. These two indicators were replaced with “*nursing*” (already included in the initial list of indicators) and “*pregnant*,” the latter being added following discussions among the three experts. Both “*nursing*” and “*pregnant*” were included in the group “*reproductive status*”. The sex and age classes were removed because the “*reproductive status*” group represents physiological states that influence porpoises’ welfare, and are inherently linked to both age and sex. Additionally, “*suckling*” and “*echelon swimming*”, which were already included in the original list of indicators, are behaviours specific to calves, and associated with a particular age category. This adjustment resulted in the removal of one indicator from the original list (–1), bringing the total number to 65. Furthermore, among the 18 additional indicators suggested by the experts’ survey, only eight were considered suitable for addition to the welfare tool, while the remaining ten were eliminated (–10 indicators), resulting in a total of 55 indicators. The eight selected indicators included “*fleeing behaviours”*, “*hygiene of fish preparation room”*, “*diversity of training session”*, “*trainer’s experience”*, “*frequency of feeding”*, “*interaction with enrichment devices”*, “*removal of food due to non-cooperation”*, and “*skin diseases”.* Additionally, further modifications were made to avoid redundancy and reduce the number of indicators. For example, “*visitors and workers disturbances”* were included within the indicator “*Unfamiliar humans’ presence”* (–1 indicator); “*Faecal Cortisol/DHEA ratio – IgA”* and “*Blowhole Cortisol/DHEA ratio – IgA”* were combined under one single indicator (–1 indicator); “*medical problems”* were included within the indicator “*general health*” (–1 indicator); “*spy hop”*, “*looking at the trainers’ office”*, and “*porpoising”* were included within the indicator “*anticipatory behaviours*” (–2 indicators); “*synchronous”* and “*contact swim”* were included within the indicator “*swimming patters*” (–1 indicator); “*breaching”* and “*jumping”* were included within the indicator “*aerial behaviours*” (–1 indicator); “*cognitive stimulation”* was removed, as its concept was already represented within “*environmental enrichment*” (–1 indicator); “*fast swimming”* was eliminated, and used as a feature of swimming speed for the different swimming patterns and behaviours included in the protocol (–1 indicator). Furthermore, during the first discussion, experts assigned each indicator to the appropriate domain(s) and categorised it as WS (Welfare Status) and/or WA (Welfare Alerting). This resulted in the final list of 46 indicators that were used to build the YFP-WAP (Table 1).

**Table 1.** Final list of 46 validated indicators for the welfare assessment of the captive Yangtze finless porpoises (YFP), selected through two rounds of surveys and four experts’ discussions, and categorised within four of the Five Domains [Nutrition (D1), Physical Environment (D2), Health (D3), and Behavioural Interactions (D4)]. Each indicator includes a definition, its contribution to YFP welfare, and its classification (category) as either welfare status (WS) or/and welfare alerting (WA). For indicators categorised as WA, the affected domain is specified in parentheses in the last column

Domain 1: Nutrition			
Indicator	Definition	Welfare Contribution	Category
Body condition scoring	Method used to assess the overall nutritional condition of an animal by visually evaluating its body shape and size.	Indicates nutritional and health status.	WS
			WA-D3
Fish intake	The amount of fish eaten by the animal during each feeding session.	Indicates the amount of fish consumed, and also affects general health.	WS
			WA-D3
Frequency of Feeding	Number of feeding sessions a day and the interval between sessions.	Influences energy balance (nutrition), behaviour, health, environmental predictability, and sense of control.	WS
			WA-D3
			WA-D4
Fish quality	Physical and nutritional characteristics of the fish used to feed the porpoises.	Indicates fish palatability and health impacts.	WS
			WA-D3

Domain 2: Physical Environment*			
Indicator	Definition	Welfare Contribution	Category
Water quality	Routine analysis of the quality of the water (Bacterial count; T°; pH).	Water quality directly impacts the health and behaviour of YFPs.	WA-D3
Pool cleaning	Process involving partially (water level 25%; water level 50%) or fully emptying the pool (water level less than 1 meter) to clean the surfaces. The porpoises are not removed from the pool.	Pool hygiene affects YFP health, appetite, and space use, with both poor cleaning or low water levels during cleaning, causing disturbance.	WA-D1
			WA-D3
			WA-D4
Hygiene of fish preparation room	Daily cleaning of the room dedicated to the preparation of the fish.	Poor hygiene may introduce harmful microorganisms and substances into food, posing health risks.	WA-D3
Moving to a different pool	One or more individuals are separated from the original group and placed in a different pool. The moving consists of removing the animals from the water and transporting them to a different pool.	Moving YFPs to another pool may influence behaviour by inducing fear and introducing excessive novelty. It can also show temporary appetite reduction, altered food intake, or changes in feeding response due to environmental novelty	WA-D1
			WA-D4
Trainer’s Experience	The trainers’ ability to effectively conduct animal training and accurately interpret the behaviour of the animals under their care.	May lead to inconsistent training, YFP frustration or unfulfilled expectations, and inadequate food rewards.	WA-D1
			WA-D4
Human-Made-Noises Disturbances	The presence of unfamiliar noise to which YFPs are not accustomed.	Disruption of the natural soundscape, potentially affecting nutrition, health, and behaviour.	WA-D1
			WA-D3
			WA-D4
Unfamiliar humans presences	Presence of construction workers or visitors around the pools or the pool-buildings.	Unfamiliar human presence is typically a disturbance for YFPs, potentially causing stress due to their sensitive nature. However, in some cases, it may provide novelty and enrichment.	WA-D1
			WA-D4
Pool size and depth	Optimal: 275 m² and at least 3.5 m depth (five individuals). + 75m² for each additional individual (EAAM regulations)	Pool size and depth determine available space, influencing behaviour.	WA-D4
Exposure to sunlight	Presence of areas with open ceiling.	Excessive sunlight may cause sunburn, while the lack of it may deprive a natural stimulus, affecting behaviour and health.	WA-D3
			WA-D4
Opportunity-of-Choice	Opportunity to select the pool, opt for isolation from other individuals, decide whether to engage in training sessions, and choose whether to play or interact with the environmental enrichment devices available.	Allows the animal to influence its environment, providing a sense of agency.	WA-D4
Diversity of training sessions	Indicates the variety of exercises taught by the trainers to the porpoises and the frequency of the exercises’ variation.	Variation in training sessions activities enhance trainer-porpoise interaction, influence behaviour, and provide cognitive stimulation.	WA-D4
Social Composition	Distribution of individual porpoises within the pools: group housing, solitary housing, and same-sex-housing.	The social environment, which can influence behaviour and contribute to positive or negative mental states depending on its appropriateness.	WA-D4
Environmental enrichment	Provision of man-made objects; opportunity to interact with trainers outside of training sessions.	An indicator of opportunity for cognitive stimulation, contributing to cognitive enrichment.	WA-D4
Removal of food due to non-cooperation	A trainer performs a time-out because the animal is not cooperating.	The fact that the animal does not receive the food affects nutrition and behaviour.	WA-D1
			WA-D4
Routine medical examinations	They consist of blood, faecal, urine, blowhole exhale samples, and ultrasound check-up.	Indicates opportunity for timely diagnosis and treatment.	WA-D3

Domain 3: Health			
Indicator	Definition	Welfare Contribution	Category
Reproductive State	Reproductive state of the porpoises: pregnant or nursing.	Pregnancy and nursing states impact the physiology, metabolism, nutrition and behaviour of porpoises.	WS
			WA-D1
			WA-D4
Tooth rakes	Presence of marks or scratches on a porpoise’s body caused by the teeth of another individual. These marks can happen during social interactions where porpoises can use their teeth to nip, rake, or scratch each other.	Fresh tooth rakes may cause pain and increase infection risk. Since YFPs do not typically engage in social play, these wounds usually indicate aggression.	WS
General health	Physiological status of the porpoises assessed by veterinary medicine check-up.	Reflects overall health, influencing nutrition and behaviour.	WS
			WA-D1
			WA-D4
Eye conditions	General status of the eyes of the porpoises.	It affects the general health and behaviour of the porpoises.	WS
			WA-D4
Skin conditions (different than tooth-rakes)	Any skin disease caused by bacterial, fungal, or viral infectious diseases. They can be more prevalent because of physiological issues or environmental, and infectious agents.	Indicates skin pathology or immune system dysfunction, potentially causing pain, itching, and behavioural changes.	WS
			WA-D4
Faecal Cortisol/DHEA ratio - IgA Blowhole Cortisol/DHEA ratio - IgA	The Cortisol/DHEA ratio is a measure of the relative levels of two hormones: cortisol and dehydroepiandrosterone (DHEA) in biological samples. Immunoglobulin A (IgA) is an antibody that can be found in various secretions, including faecal and respiratory samples.	Indicator of health that may impact behaviour and nutrition.	WS
			WA-D1
			WA-D4

Domain 4: Behavioural Interactions			
Indicator	Definition	Welfare Contribution	Category
Route tracing	Porpoise is repeatedly swimming along a predetermined path or following a specific route within its enclosure.	May indicate past or present suboptimal environmental conditions.	WS
Solitary swimming	Porpoise is swimming alone.	May indicate sub-optimal group dynamics or health issues. Effects on welfare are dependent on the combination og swimming speed and duration of the behavior.	WS
Group swimming	Coordinated behaviour displayed by a group of porpoises where the animals maintain a specific formation or pattern as they swim together.	Cohesive and affiliative behaviour, protection against threats, way to cope with stress. Effects on welfare are dependent on the swimming speed.	WS
Swimming Patterns	Synchronous swimming is a coordinated swimming behaviour exhibited by two or more porpoises swimming and breathing in synchrony with a distance of less than one body length between them. Contact Swimming is a swimming behaviour where two or more porpoises swim closely together while keeping body contact (usually using pectoral fins).	Indicates cohesion, affiliation, protection, and stress coping. Effects on welfare are dependent on the swimming speed.	WS
Directional Swimming	Porpoise is swimming in a clockwise or counter clockwise direction within their pool. This behaviour can be performed in solitary or in group formation.	May indicate past or present environmental suboptimal conditions.	WS
Aggressive Behaviours	All aggressive interactions performed by porpoises: biting, tail slapping, threatening, and chasing.	Possible indicators of social conflicts.	
Play Behaviours	All playful interactions performed by porpoises (alone or with conspecifics): object play, bubble play, and locomotor play	May indicate good health and mental state; absence could signal social, environmental, or health issues.	WS
Socio-Sexual Behaviours	All sexual activities performed by porpoises: genital-to-genital, genital-to-non genital, belly-to-belly contact, and mounting.	Serves as affiliative, playful, and reproductive behaviour.	WS
Aerial Behaviours	Jumping and breaching behaviours.	Behaviours that can be expressed in many contexts, from play to avoidance.	WS
Calf Swim	Echelon swim: a calf swims in very close proximity of its mother’s mid-lateral flank. Infant swim: a calf swim under the mother tail section, near the genital region.	Indicates strong mother-infant attachment; absence may signal abnormal maternal behaviour.	WS
Rubbing behaviour	A porpoise rubs body with elements of the environment.	Rubbing can occur for pleasure, for itchiness, or can denote behavioural issues.	WS
			WA-D3
Exploratory behaviours	A porpoise examines or interacts with elements within their environment.	Sign of mental engagement.	WS
Floating at the surface	A porpoise maintains a horizontal position with its body at the water’s surface. The tail fluke is just beneath the surface, partially submerged. The porpoise may slowly drift or rotate while floating at the surface.	May indicate a relaxed state, a sign of boredom, or illness.	WS
Behavioural diversity index	Frequency and diversity of the porpoises’ behaviour.	Denotes the quality of the physical and social environment that allow the porpoise to engage in a range of behaviours.	WS
Evasion behaviours	Porpoises avoid social interactions (with human or conspecifics) with change of direction, U turn, fast movement away, or porpoising.	Indicates distress, negative experiences, and survival response.	WS
Interaction with Enrichment Devices	A porpoise interacts with the objects or devices made available to them.	Indicates engagement and partial fulfilment of mental stimulation needs.	WS
Latency to come to the trainer	Time interval, usually measured in minutes, between the trainer’s arrival at the pool edge and the porpoise approaching the trainer.	Indicates the motivation of the animal to interact with the trainer and/or to eat.	WS
			WA-D1
			WA-D3
Breaking the interaction with trainer	Number of times the porpoise leaves its trainer’s location during a training session.	Indicates the motivation of the animal to interact with the trainer and/or to eat.	WS
			WA-D1
			WA-D3
Response to the trainer	Quality of the porpoise response to the trainer’s training cues.	Indicates the motivation of the animal to interact with the trainer and/or to eat.	WS
			WA-D1
			WA-D3
Anticipatory behaviours	All behaviours performed by the porpoises that indicate anticipation of an event: looking at the trainers’ office, spy-hop, and porpoising.	Signs of anticipation, usually deemed to be linked to positive mental states associated with previous experiences.	WS
Suckling	A porpoise calf is feeding on its mother’s milk.	Provides maternal bonding, nutrition, and health benefits.	WS
			WA-D1
			WA-D3

* The domain Physical Environment includes only WA indicators.

#### Contribution of the indicators to the four physical domains, mental states, valence, and intensity level

During the second, third, and fourth discussions, the experts determined indicators’ valence (welfare compromise or welfare enhancement), intensity levels, and associated mental states. Among the 46 indicators, some were relevant to multiple domains and, depending on the context, could be categorised as WC (Welfare Compromise) and WE (Welfare Enhancement), WS (Welfare Status) and/or WA (Welfare Alerting). Specifically, all 15 indicators in Domain 2 – Physical Environment were categorized as WA for both their primary domain and other domains; 18 WS indicators were assigned exclusively to their primary domain (16 in domain 4 – Behavioural Interactions, and 2 in domain 3 – Health); 14 indicators were classified as WS on their primary domain, and WA on another (Table 2). For example, “*body condition scoring (BCS)*” was recognised as a WS indicator within domain 1 – Nutrition, and as WA in domain 3 – Health. Additionally, an ideal BCS was classified as WE, whereas a skinny and obese body conditions were classified as WC in both the Nutrition and Health domains. This illustrates how each indicator can impact upon an animal’s welfare differently depending on the domain in which it is applied, its valence, and the category to which it belongs. Therefore, each of the original 46 indicators could be assigned to multiple domains, increasing the total number of entries in the final protocol. This approach enabled the development of a more comprehensive tool, the YFP-WAP (Yangtze Finless Porpoises-Welfare Assessment Protocol), that includes a total of 150 indicators (Supplementary material; S-3).

**Table 2.** Total number of indicators included in each domain (1 = Domain 1 -Nutrition; 2 = Domain 2-Physical Environment; 3 = Domain 3- Health; 4 = Domain 4 - Behavioural Interactions) for the final version of the YFP-WAP with the percentage (% in parentheses beside the total number of indicators) by domain, valence (Welfare Compromise - WC; Welfare Enhancement - WE), and intensity (Low-Mild-High), and indicator type (Status-Alerting). Different indicators have varying intensity level depending on the condition observed. In the table the highest intensity level for each indicator was reported

Domain	Indicator Type		Valence		Intensity Level			Total: number and (%)
	Status	Alerting	WC	WE	Low	Mild	High	
1	10 (6.7)	16 (10.7)	17 (11.3)	9 (6)	4 (2.7)	9 (6)	13 (8.7)	26 (17.3)
2	0	24 (16)	13 (8.7)	11 (7.3)	4 (2.7)	4 (2.7)	16 (10.7)	24 (16)
3	8 (5.3)	28 (18.7)	22 (14.7)	14 (9.3)	16 (10.7)	17 (11.3)	3 (2)	36 (24)
4	35 (23.3)	29 (19.3)	27 (18)	37 (24.7)	11 (7.3)	23 (15.3)	30 (20)	64 (42.7)
Total number and (%)	53 (35.3)	97 (64.7)	79 (52.7)	71 (47.3)	35 (23.3)	53 (35.3)	62 (41.4)	150 (100)

Furthermore, confidence scores were assigned to each indicator’s mental state and intensity level. Indicators’ intensity level (median, M_intensity_ = 2) and mental states (median, M_intensity_ = 2) obtained similar confidence scores across all domains, except for domain 3 – Health where the mental state scored lower (median, M_intensity_ = 1). Likewise, the indicators’ intensity levels and associated mental states for either the WE and WC, as well as for welfare status (WS) and welfare alerting (WA) received similar confidence scores (median, M_intensity_ = 2). Since each indicator was assigned more than one mental state, this allowed calculation of an average confidence score. Indicators that obtained the highest average confidence score (> 2.5) for their attributed mental states were four in D1, four in D2, one in D3, and nine in D4 (for assigned mental states, see Supplementary material; S-3). Conversely, intensity levels were represented by single values for each indicator, therefore an average value could not be calculated. The indicators that obtained the highest confidence score (3) for their intensity level, within each domain, were four in D1, ten in D2, four in D3, and eight in D4 (Table 3, and refer to Supplementary material; S-3 for assigned valence and category).

**Table 3.** List of indicators that achieved the highest average confidence score for mental states, and the highest confidence score for intensity level. The √ symbol denotes indicators that met the highest confidence score threshold (> 2.5 for mental states; 3 for intensity level), while × indicates those that did not. Confidence scores for mental states represent an average value across multiple states, whereas intensity levels were assigned a single score per indicator. Indicators are categorised within each domain: D1 (Nutrition), D2 (Physical Environment), D3 (Health), and D4 (Behavioural Interactions)

Domain	Indicator	Highest average confidence score in mental states (> 2.5)	Highest confidence score in intensity level (3)
D1	Body Condition Scoring	**√**	**√**
	Fish intake	**√**	**√**
	Frequency of feeding	**√**	**√**
	Fish quality	**√**	**√**
D2	Opportunity of choice	**√**	**√**
	Moving to a different pool	**√**	**√**
	Pool size and depth	**√**	×
	Diversity of training session	**√**	**√**
	Hygiene of fish preparation room	×	**√**
	Environmental enrichment	×	**√**
	Social composition	×	**√**
	Pool cleaning	×	**√**
	Human-made-noise disturbances	×	**√**
	Exposure to sunlight	×	**√**
	Removal of food due to non-cooperation	×	**√**
D3	General health	**√**	**√**
	Routine medical examination	×	**√**
	Eye conditions	×	**√**
	Skin conditions	×	**√**
D4	Play behaviours	**√**	**√**
	Socio-sexual behaviours	**√**	×
	Exploratory behaviours	**√**	×
	Latency to come to the trainer	**√**	×
	Breaking the interaction with the trainer	**√**	×
	Response to the trainer	**√**	×
	Echelon swim	**√**	**√**
	Anticipatory behaviours	**√**	×
	Interaction with enrichment devices	×	**√**
	Group swimming	×	**√**
	Swimming patterns	×	**√**
	Fleeing behaviours	×	**√**
	Behavioural Diversity Index	×	**√**
	Suckling	**√**	**√**

## Discussion

The current study aimed to develop a welfare assessment tool for captive Yangtze finless porpoises based on the FDM. A total of 46 indicators were selected and used to build the Yangtze Finless Porpoise-Welfare Assessment Protocol (YFP-WAP) that, in its final version, comprises 150 welfare indicators. The framework includes three survival-critical domains (Nutrition, Health, and Physical Environment), and one situation-related domain (Behavioural Interactions). The outcomes, positive or negative, for each domain, are used to infer the animal’s specific mental state (frustration, hunger, pain, thirst) in the fifth domain (Mellor *et al.* 2020; Raciatti et al. 2022).

### Validation of the indicators to develop the YAP-WAP

The current protocol used expert opinion to validate a list of indicators for the welfare assessment of the YFP. In general, the validation of an animal welfare assessment tool that involves expert opinion might introduce some biases (Hampton *et al.* 2016; Buddle *et al.* 2018). For example, the panel members could show subjectivity to the process, with some experts preferring certain fields compared to others (Fraser *et al.* 1997; Collins *et al.* 2018; Sherwen *et al.* 2018; Johnson *et al.* 2019). For this reason, it is important to clearly outline how experts are recruited and how the validation process is conducted. At present, only a few studies have provided clarity on this process (Beausoleil *et al.* 2016; Sherwen *et al.* 2018; Allen *et al.* 2019; Baker *et al.* 2022; De Ruyver *et al.* 2023; Serres *et al.* 2024). Specifically, the method by Serres *et al.* (2024) was used as a model to develop a precise protocol for the current study, which described the expert opinion survey in detail, and the recruitment of experts with diverse areas of expertise — such as cetacean biology, conservation, animal welfare, veterinary medicine, and professionals working with cetaceans in human care, including YFP husbandry and conservation — for the validation process. The selection of experts with a broad range of expertise ensured that the indicators chosen were both practical and relevant to be used by aquarium staff and researchers. Four different panels were set up in order to reduce the biases related to experts’ subjectivity: two groups for the questionnaire surveys, one group for the four discussions, and a different group of three experts for the blind review. Even though some experts involved in the two surveys were only “*somewhat familiar*” with either animal welfare or cetaceans, their contributions were still considered valuable in the selection of YFP-WAP indicators. Moreover, the diverse expertise within the two panels helped mitigate the potential influence of individuals with limited familiarity in either field, ensuring a more balanced and comprehensive selection process for the indicators. Ensuring a balanced and diverse panel can minimise subjective biases, reduce the risks related to the wrong elimination of indicators, and improve the reliability of the results. This approach allows for a more thorough evaluation of the welfare indicators by incorporating multiple perspectives, ensuring that a wide range of behaviours and considerations are not overlooked (Sandøe *et al.* 2019). Moreover, the decision to use a relatively small number of experts (57 in the first survey and 59 in the second) for the validation process was based on the concern that involving a larger group could introduce additional biases by increasing the risk of including individuals without specific expertise in animal welfare, cetaceans, or YFPs. This approach minimises the likelihood of diluting the insights with opinions from those lacking the necessary specialised knowledge, which could otherwise compromise the accuracy and applicability of the validation process.

### Indicators inclusion within the four domains

The YFP-WAP shows a disparity regarding the number of indicators included in the category WA compared to WS within each domain. Specifically, the protocol includes more welfare-alerting indicators than welfare-status indicators, indicating that the resulting scores should be interpreted with caution. Notably, welfare-alerting indicators signal the risk that a condition could arise, rather than having a direct, proven link to an animal’s mental state. Despite that, welfare-alerting indicators might provide inferred mental states when it is assumed that the object of the alert would actually occur. For this reason, they can sometimes be used as proxies for animal-based measures when none are available. In addition, alerting indicators may help not only to identify animals with poor welfare, but also those whose welfare is already declining (EFSA 2012; Harvey *et al.* 2020). Examples of alerting indicators that can provide crucial information on porpoises’ welfare is the “*social composition*”. It is well established that social dynamics among cetaceans, along with the sex and age classes of individuals within the group, play a crucial role in maintaining group stability and, consequently, in ensuring the welfare of each animal (Waples & Gales 2002; Johnson & Norris 2013). Furthermore, welfare compromise indicators are the second most represented in the YFP-WAP, followed closely by welfare enhancement indicators, with only a nine-indicators difference between them. Having a very close number of indicators for welfare compromise and enhancement, it does not automatically mean that the final outcome of the framework might be “more balanced”. However, it is important to consider the weight or severity that each indicator plays on the welfare of the porpoises. Nevertheless, having a very similar number for welfare enhancement and compromise indicators could still allow for more sensitive detection of issues that positively or negatively impact the animals leading to a prompter intervention.

The number of indicators within each domain also varied. For example, Domain 2 –Physical environment has the lowest number of indicators followed by Domain 1 – Nutrition, Domain 3 – Health, while Domain 4 – Behavioural Interactions has the highest number of indicators. Domain 4 also contains the highest number of welfare status indicators (animal-based indicators), which suggests that this domain may provide better insights into the welfare of YFP individuals compared to the other domains, as animal-based measures are considered to be closely linked to animal welfare (Capdeville & Veissier 2001; Whay *et al.* 2003; Winckler *et al.* 2003). Nevertheless, the unequal distribution of indicators across domains might lead to some bias, with certain domains having a greater influence on the final output than others. However, this effect may be mitigated by the fact that indicators that are welfare status in one domain are also included in other domains as alerting indicators, with their scores reflecting their specific impact within each domain. As a result, some indicators may have a greater influence on the final outcome depending on their associated domain and scores. This approach helps minimise bias caused by variations in the number of indicators across domains. Moreover, the type of scoring system (which will be developed in the second phase of the current project) can also have great impact on how the indicators of each domain influence the final welfare score.

### Attribution of confidence scores

In the current study, experts provided confidence scores for mental states and intensity levels for each indicator to reflect their level of uncertainty regarding the impact each indicator has on the welfare of the YFP. It is interesting that both the indicators’ mental states and intensity level across all domains received a medium confidence score (2), suggesting that the experts were fairly confident in both the mental state the indicator could cause, and the level of impact each indicator could have on the welfare of individual porpoises (Baker *et al.* 2022). In addition, this confidence score might also stem from the experts’ familiarity with the effects of different conditions on the welfare of cetaceans under human care. On the other hand, a lower confidence score (1) was assigned to the indicators’ mental states of domain 3 – Health, which could represent the greater difficulty in inferring mental states for health indicators such as tooth rakes and reproductive state (Browning 2022b).

Therefore, mental states must be inferred from observable indicators of the animal’s physical and psychological states, which is why animal-based measures are used for this purpose (Mellor *et al.* 2020; Browning 2022a). For instance, within the YFP Welfare Assessment Protocol (YFP-WAP), play behaviours (WS for Domain 4 – Behavioural Interactions) received high confidence scores for both mental state and intensity levels. Play is considered an important cognitive activity, and its absence could negatively affect the psychological well-being of the animal (Boissy *et al.* 2007). However, it is important to emphasise that a single observation of a lack of play activities is insufficient to raise a concern. Therefore, repeated observations must be considered to provide a more accurate assessment of the potential issues related to the absence of play behaviours. Moreover, in Domain – 4, besides all welfare indicators that specifically address the dynamics of the interaction among YFP conspecifics, interactions with trainers are also considered. Precisely, the indicators such as “*latency to come to the trainer*”, “*breaking the interaction with the trainer*”, and “*response to the trainer*” (to training cues), which received high average confidence scores for their associated mental states, have been regarded as crucial for the welfare of cetaceans under human care, and thus considered critical management tools (Ramirez 2012; Clegg *et al.* 2015). For example, observations have shown that dolphins respond positively to trainers’ behavioural requests when these are rewarded with positive social interactions rather than food alone. This supports the view that cetaceans benefit from and enjoy interactions with their trainers (Perelberg & Schuster 2009; Platto & Serres 2023). Additionally, the responsiveness of dolphins to the presence of trainers, and the activities proposed to them has been used as an indicator of welfare. Precisely, dolphins that are less responsive to the trainers’ request often show signs of compromised well-being (Clegg *et al.* 2018, 2019; Delfour *et al.* 2020). Moreover, all indicators classified under Domain-1 (Nutrition) achieved the highest average confidence scores for both mental states and intensity levels. It is well known that nutrition plays a crucial role not only in maintaining physical health, but also in influencing behavioural outcomes (Clegg *et al.* 2018, 2019; Delfour *et al.* 2020). Regular assessment and adjustment of nutritional strategies based on observed health and behaviour can further enhance the well-being of these animals.

Furthermore, the panel of experts validated physiological indicators such as the faecal/blowhole cortisol:DHEA ratio, and mucosal IgA for the YFP-WAP. The use of the cortisol:DHEA ratio as an indicator of animal welfare has only recently been developed for cetaceans under human care (Gundlach *et al.* 2018; Whitham *et al.* 2020; Lauderdale *et al.* 2021; Miller *et al.* 2021b; Reckendorf *et al.* 2021), which could also explain the medium confidence score given to this indicator by the panel of experts. DHEA acts as an antagonist to cortisol, increasing in response to acute stress, and decreasing during chronic stress (Guilliams & Eduards 2010; Fustini *et al.* 2017; Kamin *et al.* 2017). These physiological indicators are often used in association with behavioural indicators such as behavioural diversity index (BDI), and route tracing. For example, Miller *et al.* (2021b) found an inverse relationship between cortisol:DHEA ratio levels and the BDI, providing further evidence that these indicators together may be valuable for assessing the welfare of cetaceans under human care (Hall *et al.* 2021). On the other hand, mucosal IgA has been studied primarily in the context of domestic animals’ welfare. In cetacean species, IgA has been examined mainly at the serum level (Travis *et al.* 1972a,b; Murata *et al.* 2004; Nollens *et al.* 2009; Ruiz *et al.* 2009). In general, the primary function of mucosal IgA is to prevent microorganisms from interacting with or penetrating the mucosal epithelium, thereby maintaining a balanced gut microbiota (Corthesy 2013). Mucosal IgA is also affected by stress through the hypothalamic-pituitary-adrenal (HPA) axis. For example, prolonged stress is known to suppress IgA secretion at the mucosal level, explaining how chronic stress can impact physical health and contribute to disease in animals (Pacella *et al.* 2013). Mucosal IgA has never been assessed in YFP. Currently, SP and her team have identified mucosal IgA in YFPs’ faecal samples, with research still ongoing. Even though knowledge about the interaction between IgA and stress hormones in cetaceans at muscosal level is not yet well established, experts have still assigned a medium confidence score to this indicator, possibly due to its perceived importance in assessing animal welfare.

### Advantages and limitations of using the YFP-WAP

There is a growing use of the FDM for assessing the welfare of wildlife under human care (Clegg *et al.* 2015; Kagan *et al.* 2015; Sherwen *et al.* 2018; Baumgartner *et al.* 2024). For example, WAZA’s animal welfare strategy recommends that zoos and aquaria apply the FDM to systematically assess animal welfare (Mellor 2015b). In the case of the YFP-WAP, it is important to remember that a single assessment of a YFP individual with the framework could be biased by seasonal changes in physiology, behaviour, sexuality, social interactions, and inappetence which is quite common in cetaceans under human care (Wells 2009). Implementing a longitudinal approach with multiple assessments during periods of atypical conditions are recommended which would mitigate potential biases and obtain a clearer picture of the individuals’ welfare and detect potential alerting changes (Botreau *et al.* 2007a,2007b). Furthermore, the YFP-WAP is not designed to classify welfare as simply a good or a bad situation. Instead, it serves as a tool for conducting systematic, structured, and comprehensive assessments of animal welfare, focusing on indicators of welfare compromise and enhancement. Precisely, the YFP-WAP (based on the FDM) allows attention to be focused upon areas of concern, guiding the implementation of solutions, including ways of promoting positive welfare states in porpoises (Yeates & Main 2008; Littin *et al.* 2014; Mellor 2015a,b,c; Mellor & Beausoleil 2015; Mellor *et al.* 2020).

Moreover, while the number of experts involved in validating the indicators might seem limited, the selected experts were chosen for their specialised knowledge in cetacean welfare and YFP. This targeted expertise enhances the reliability of the results, as it ensures that the framework’s validation is conducted by individuals with relevant experience. Including a broader range of experts from unrelated fields might introduce biases due to a lack of specific knowledge regarding cetacean biology and welfare. Additionally, the use of three distinct types of panels — survey, discussion, and blind review — has helped minimise biases, such as the potential for incorrect elimination of indicators due to expert subjectivity. This multi-faceted approach ensures a more balanced and accurate evaluation of the indicators, reinforcing the framework’s robustness and credibility in assessing welfare. Therefore, it is essential to consider that a well-rounded welfare assessment framework such as the YFP-WAP not only identifies immediate needs but also aids in the long-term monitoring and improvement of animal welfare.

### Animal welfare implications

The framework supports continuous enhancement of care practices and contributes to a deeper understanding of porpoises’ well-being under human care through integrating insights from specialised experts and employing rigorous validation processes. As such, the YFP-WAP is extensive in its scope, encompassing a broad range of indicators and providing a comprehensive view of the welfare status of the observed individuals. This holistic approach ensures that the framework remains robust and attuned to the ever-changing needs of the animals. Although the protocol was developed specifically for Yangtze finless porpoises, it is highly versatile and would require only minor adjustments, taking into account species-specific environmental and behavioural needs, to also be applicable for marine subspecies of finless porpoises under human care. While certain physiological or ecological differences may necessitate species-specific modifications (e.g. variations in habitat requirements or social structures), the core framework can also be applicable to a broad range of cetaceans in captivity, ensuring that their physical and psychological needs are comprehensively and appropriately addressed. It is important to consider that a well-designed welfare assessment tool should incorporate flexibility to accommodate the variability of the environmental and management conditions of the species in question. Also, it should include mechanisms for ongoing feedback and refinement based on practical experience and emerging research.

### Future development of the YFP-WAP

The development of a welfare assessment tool involves several critical steps (Botreau *et al.* 2007a,b; Hampton *et al.* 2023). In this paper, the initial steps have been clearly outlined, leading to the validation of the indicators that constitute the YFP-WAP. The next phase involves establishing a suitable scoring system for the protocol that ensures systematic and reliable welfare assessments across various conditions. The final objective is to obtain a welfare assessment tool adaptable and applicable not only to YFP under human care, but also to all finless porpoises of the genus *Neophocaena* in captivity. Further steps are still required to finalise the protocol’s readiness for practical application. These include conducting validation trials to evaluate the tool’s feasibility in real-world settings. Such trials will test the practicality of the protocol, ensuring that it performs effectively under diverse conditions and provides actionable insights into animal welfare. By integrating these elements, the protocol can enhance its utility and reliability, ultimately contributing to improved welfare outcomes for captive porpoises and other cetacean species under human care. Moreover, animals, especially those under human care, do not exist in isolation, so it is important to approach the assessment of their welfare in a more holistic, one-welfare framework, where the individual animal is viewed in connection with its surrounding physical environment and human interactions.

## Supporting information

Platto et al. supplementary material Platto et al. supplementary material

## Supplementary material

The supplementary material for this article can be found at http://doi.org/10.1017/awf.2025.19.
