# Supplementary material for: Validation of indicators for the welfare assessment of captive Yangtze finless porpoises (Neophocaena asiaeorientalis asiaeorientalis)
Source: Anim Welf. 2025 May 21;34:e31. doi: 10.1017/awf.2025.19 (PMC12170231; doi:10.1017/awf.2025.19)
Supplement: Platto et al. supplementary material [file S0962728625000193sup001.pdf]

**Panel Review Tables and Figures**

**S-1**. Experts' characteristics involved in the two rounds of surveys (numbers indicate % of experts' response) for Panel 1 (total of 30 responses) and for Panel 2 (total of 33 responses).

| **Characteristic** | **Level** | **Panel 1** | **Panel 2** |
|---|---|---|---|
| Education | PhD | 46.3 | 63.6 |
| | Master | 30.0 | 24.2 |
| | Bachelor | 23.3 | 12.1 |
| Expertise | Animal Behavior | 56.7 | 48.5 |
| | Animal Welfare | 56.7 | 57.6 |
| | Veterinary Medicine | 40.0 | 42.4 |
| | Conservation Biology/Ecology | 26.7 | 30.3 |
| | Animal Sciences | 20.0 | 15.2 |
| | Other | 3.0 | 15.2 |
| Familiarity with animal welfare | Extremely familiar | 16.7 | 27.3 |
| | Very familiar | 36.7 | 24.2 |
| | Familiar | 23.3 | 18.2 |
| | Somewhat familiar | 10.0 | 30.3 |
| | Not at all familiar | 13.3 | 0.0 |
| Familiarity with cetaceans | Extremely familiar | 16.7 | 39.4 |
| | Very familiar | 30.0 | 36.4 |
| | Familiar | 20.0 | 18.2 |
| | Somewhat familiar | 23.3 | 12.1 |
| | Not at all familiar | 10.0 | 0.0 |
| Knowledge of YFP | Excellent | 13.3 | 21.2 |
| | Good | 26.7 | 18.2 |
| | Fair | 23.3 | 18.2 |
| | Poor | 30.0 | 24.2 |
| | Very poor | 6.0 | 18.2 |

**S-2**. Experts' opinion regarding the validity of the 49 indicators considered for the development of the welfare assessment tool for the YFP. The red dot line represents the limit (60% of "Yes" answers) for the inclusion of the indicators in the welfare assessment framework.

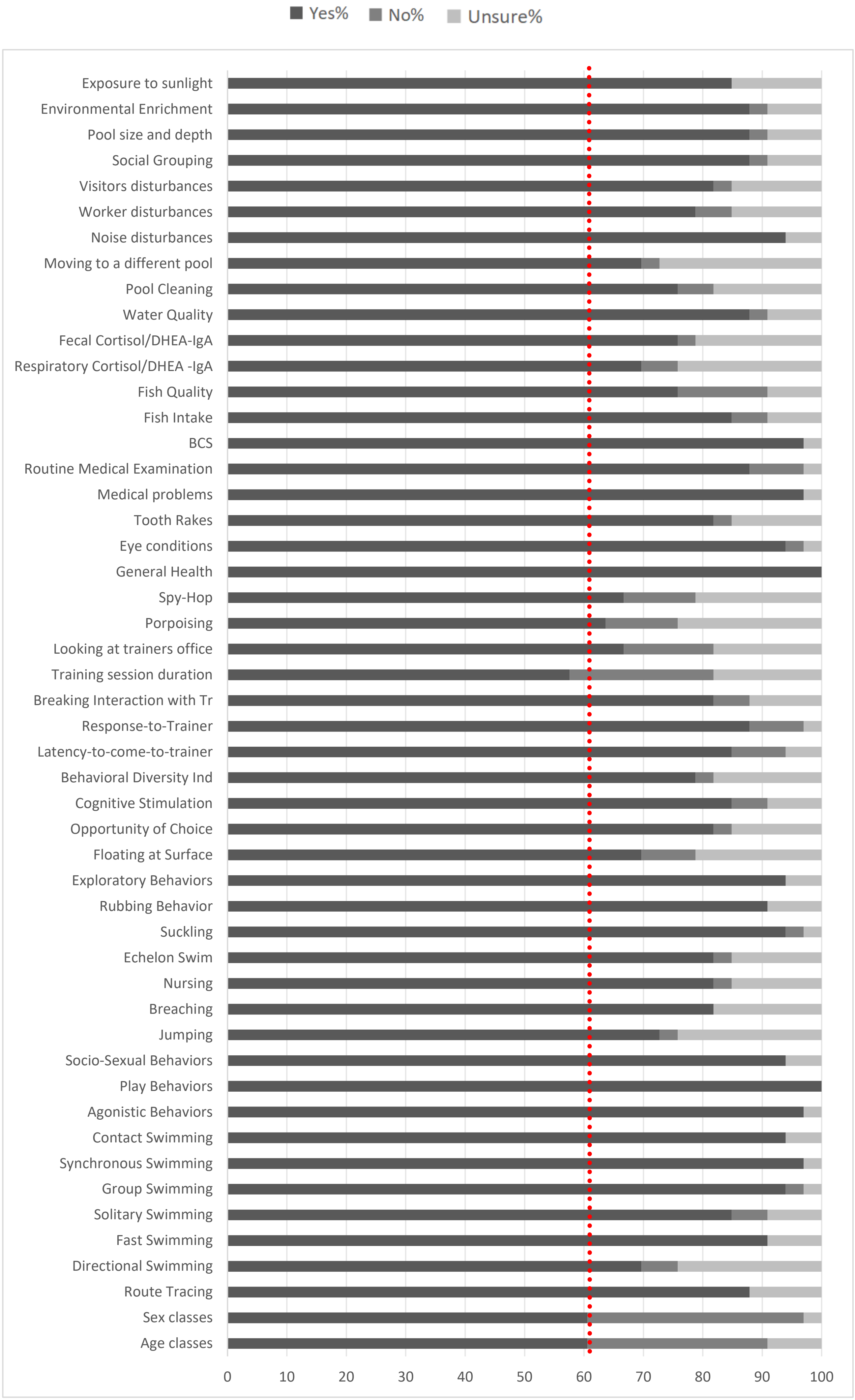
Yes%
No%
Unsure%
Exposure to sunlight
Environmental Enrichment
Pool size and depth
Social Grouping
Visitors disturbances
Worker disturbances
Noise disturbances
Moving to a different pool
Pool Cleaning
Water Quality
Fecal Cortisol/DHEA-IgA
Respiratory Cortisol/DHEA -IgA
Fish Quality
Fish Intake
BCS
Routine Medical Examination
Medical problems
Tooth Rakes
Eye conditions
General Health
Spy-Hop
Porpoising
Looking at trainers office
Training session duration
Breaking Interaction with Tr
Response-to-Trainer
Latency-to-come-to-trainer
Behavioral Diversity Ind
Cognitive Stimulation
Opportunity of Choice
Floating at Surface
Exploratory Behaviors
Rubbing Behavior
Suckling
Echelon Swim
Nursing
Breaching
Jumping
Socio-Sexual Behaviors
Play Behaviors
Agonistic Behaviors
Contact Swimming
Synchronous Swimming
Group Swimming
Solitary Swimming
Fast Swimming
Directional Swimming
Route Tracing
Sex classes
Age classes
0
10
20
30
40
50
60
70
80
90
100

**S-3.** Welfare alerting (WA) and welfare status (WS) indicators contributing to Domain 1 (Nutrition -D1), Domain 2 (Environment- D2), Domain 3 (Health- D3), and Domain 4 (Behavioral Interactions -D4), and the associated inferred mental states and intensity levels for Yangtze Finless porpoises. Numbers in parentheses are confidence scores attributed by experts to mental states and intensity levels based on their knowledge and experience. Numbers in parentheses in the conditions column of Domain 2 are the domains that the specific indicator is affecting as WA indices.

| Domain | Valence | Category | Indicator | Conditions | Intensity Level/ Confidence Scores | Mental state/Confidence Scores |
|---|---|---|---|---|---|---|
| **D1** | **Welfare Enhancement** | **Welfare Status** | Body Condition | Ideal | +++ (3) | Comfort (2)<br>Satiate (3) |
| | | | Fish intake | Optimal | +++ (3) | Satiate (3) |
| | | | Frequency of feeding | Optimal | +++ (3) | Satiate (3)<br>Satisfied (3)<br>Motivation to eat (3) |
| | | | Fish quality | Good | +++ (3) | Satiate (3)<br>Satisfied (3)<br>Motivation to eat (3) |
| | | **Welfare Alerting** | Trainer's Experience | Experienced | + (2) | Motivated to eat (2)<br>Comfort (0) |
| | | | Latency to come to the trainer | 0-1 minute | ++ (2) | Satiated (3)<br>Comfort (3) |
| | | | Breaking Interaction with the trainer | 0-3 times | ++ (2) | Satiated (3)<br>Comfort (3) |
| | | | Response to the trainer | Approach/good motivation | ++ (2) | Satiated (3)<br>Comfort (3) |
| | **Welfare Compromise** | **Welfare Status** | Body Condition | Skinny | D (3) | Hunger (3) |
| | | | | Overweight | B (3) | Gluttonous (1) |
| | | | Fish intake | Sub-optimal | D (3) | Hungry (3)<br>Loss of appetite (1) |
| | | | Frequency of Feeding | Sub-optimal | D (1) | Hungry (3)<br>Discomfort (2)<br>Weakness (1) |

| | | | | | | |
|---|---|---|---|---|---|---|
| | | | Fish Quality | Poor | D (3) | Hungry (2)<br>Disgusted (2)<br>Unmotivated to eat (2) |
| | | **Welfare Alerting** | Pool Cleaning | Presence | D (3) | Unmotivated to eat (2)<br>Hungry (1) |
| | | | Moving to a different pool | Presence | D (3) | Unmotivated to eat (2)<br>Hungry (0) |
| | | | Trainer's Experience | Not-experienced | B (2) | Lack motivation to eat (2) |
| | | | Humans-made-noises disturbance | Presence | C (3) | Unmotivated to eat (2) |
| | | | Unfamiliar human presence | Presence negative | B (1) | Unmotivated to eat (1) |
| | | | Removal of food due to non-cooperation | Presence | C (3) | Hungry (2) |
| | | | Reproductive State | Pregnant | B (2) | Unmotivated to eat (2) |
| | | | | Nursing | B (2) | Unmotivated to eat (2) |
| | | | Fecal/Blowhole Cortisol/DHEA ratio | ratio > 0 | D (2) | Unmotivated to eat (2) |
| | | | Fecal/Blowhole IgA levels | Below minimum requirements | D (2) | Unmotivated to eat (2) |
| | | | Latency to come to the trainer | over 1 minute | C (2) | Hungry (1) |
| | | | Breaking interaction with the trainer | more than 3 times | C (2) | Hungry (1) |
| | | | Response to the trainer | No approach | C (2) | Hungry (1) |
| | | | | Approach-low motivation | C (2) | Not satiated (1) |

| Domain | Valence | Category | Parameter | Conditions | Intensity Level/ confidence scores | Mental state and confidence scores |
|---|---|---|---|---|---|---|
| **D2** | **Welfare Enhancement** | **Welfare Alerting** | Water quality | Acceptable (D3) | ++ (2) | Comfort (0) |
| | | | Pool Cleaning | Presence (D3) | ++ (2) | Comfort (0) |
| | | | Hygiene of fish preparation room | Good (D3) | + (3) | Comfort (0) |
| | | | Pool size and depth | Optimal (D4) | + (2) | Comfort (1) |
| | | | Exposure to sunlight | Presence Positive (D3) | + (2) | Comfort (1)<br>Pleasure (0) |
| | | | | Presence Positive (D4) | +++ (2) | Active (1) |

| | | | | | | |
|---|---|---|---|---|---|---|
| | | | Environmental enrichment | Presence (D4) | +++ (3) | Stimulated (3)<br>Curious (2)<br>Interactive (1)<br>Engaged (1)<br>Playful (1)<br>Sense of agency (2) |
| | | | Trainer's experience | Experienced (D1) | + (2) | Motivated to eat (2)<br>Comfort (0) |
| | | | | Experienced (D4) | + (2) | Engaged (3)<br>Playful (1)<br>Cooperative (2) |
| | | | Unfamiliar human presence | Presence positive (D4) | + (1) | Curious (2)<br>Interactive (2)<br>Playful (2) |
| | | | Opportunity of Choice | Presence (D4) | +++ (3) | In control (3)<br>Stimulated (3)<br>Satisfied (2) |
| | | | Diversity of training sessions | Presence (D4) | +++ (2) | Stimulated (2)<br>Curious (0)<br>Engaged (1)<br>Playful (0) |
| | | | Social Composition | Social grouping Positive (D4) | +++ (3) | Social support (3)<br>Affectionate (2)<br>Playful (2)<br>Sexually fulfilled (1)<br>Comfort (1) |
| | | | | Same-sex-grouping-Females (D4) | + (1) | Social support (2)<br>Affectionate (2)<br>Playful (1) |
| | **Welfare Compromise** | **Welfare Alerting** | Water quality | Unacceptable(D3) | D (2) | Discomfort (1) |
| | | | Pool cleaning | Absence (D3) | C (2) | Discomfort (1) |
| | | | | Presence (D1) | D (3) | Unmotivated to eat (2)<br>Hungry (1) |

| | | | | | | |
|---|---|---|---|---|---|---|
| | | | | Presence (D4) | D (3) | Fear (3)<br>Uncomfortable (3)<br>Distressed (3)<br>Vigilance (3)<br>Insecurity (1)<br>Panic (1) |
| | | | Hygiene of fish preparation room | Bad (D3) | D (3) | Discomfort (2)<br>Gastrointestinal discomfort (2) |
| | | | Moving to a different pool | Presence (D1) | D (3) | Unmotivated to eat (2)<br>Hungry (0) |
| | | | | Presence (D4) | D (3) | Lost control (3)<br>Panic (3)<br>Vigilant (2)<br>Fear (3)<br>Feeling lack of social support (2)<br>Distressed (3) |
| | | | Pool size and depth | Sub-Optimal | D (2) | Discomfort (3)<br>Boredom (3)<br>Frustration (2) |
| | | | Exposure to sunlight | Absence (D3) | C (1) | Discomfort (1) |
| | | | | Absence (D4) | D (1) | Depressed (1) |
| | | | | Presence Negative (D3) | D (2) | Discomfort (2)<br>Pain (2)<br>Malaise (1) |
| | | | | Presence Negative (D4) | D (2) | Annoyed (1)<br>Frustration (1) |
| | | | Environmental enrichment | Absence (D4) | D (3) | Frustration (1)<br>Bored (2) |
| | | | Trainer’s experience | Not-experienced (D1) | B (2) | Lack motivation to eat (2) |
| | | | | Not-experienced (D4) | B (2) | Frustrated (2)<br>Bored (2) |

| | | | | | | |
|---|---|---|---|---|---|---|
| | | | | | | Loss of control (1) |
| | | | Human-made-noise disturbances | Presence (D1) | C (3) | Unmotivated to eat (2) |
| | | | | Presence (D4) | D (3) | Agitated (2)<br>Annoyed (1)<br>Vigilant (3)<br>Discomfort (1)<br>Loss of control (2)<br>Fear (1) |
| | | | | Presence (D3) | B (3) | Acoustic discomfort (2)<br>Disorientation (1) |
| | | | Unfamiliar human presence | Presence negative (D1) | B (1) | Unmotivated to eat (1) |
| | | | | Presence negative (D4) | B (1) | Fear (1)<br>Anxiety (0)<br>Annoyance (2)<br>Hyper-vigilance (2)<br>Loss of control (1) |
| | | | Opportunity of Choice | Absence (D4) | D (3) | Frustration (2)<br>Bored (2) |
| | | | Diversity of training sessions | Absence (D4) | D (2) | Bored (3)<br>Unmotivated to participate (3) |
| | | | Group housing | Solitary housing-Presence (D4) | D (3) | Lonely (3)<br>Apathetic (1)<br>Frustrated (1)<br>Depressed (2)<br>Bored (2) |
| | | | | Same-sex-housing Males (D4) | B (1) | Sexually frustrated (2) |
| | | | | Same-sex-housing Females (D4) | B (1) | Sexually frustrated (0) |
| | | | | Negative (D4) | D (3) | Discomfort (2)<br>Withdrawn (2)<br>Fear (2)<br>Frustration (2) |

| | | | | | | |
|---|---|---|---|---|---|---|
| | | | | | | Anxiety (2) |

| Domain | Valence | Category | Parameter | Conditions | Intensity Level/ confidence scores | Mental state and confidence scores |
|---|---|---|---|---|---|---|
| **D3** | **Welfare Enhancement** | **Welfare Status** | General Health | Adequate | ++ (3) | Vitality (3)<br>Comfort (2) |
| | | **Welfare Alerting** | Water quality | Good | ++ (2) | Comfort (0) |
| | | | Pool Cleaning | Presence | ++ (2) | Comfort (0) |
| | | | Hygiene of fish preparation room | Good | + (3) | Comfort (0) |
| | | | Exposure to sunlight | Presence positive | + (2) | Comfort (1) |
| | | | Frequency of feeding | Optimal | ++ (1) | Energetic (2)<br>Comfort (2) |
| | | | Fish quality | Good quality | ++ (3) | Gastrointestinal comfort (2)<br>Vitality (2) |
| | | | Body condition scoring | Ideal | + (3) | Comfort (2)<br>Vital (2) |
| | | | Fish intake | Optimal | +++ (3) | Vitality (2)<br>Comfort (0) |
| | | | Suckling | Presence | +++ (3) | Energetic (1)<br>Active (1) |
| | | | Latency to come to the trainer | (0-1 minute) | ++ (2) | Energetic (3)<br>Vital (3) |
| | | | Breaking interaction with the trainer | (0-3 times) | ++ (2) | Energetic (3)<br>Vital (3) |
| | | | Response to the trainer | Approach-good motivation | ++ (2) | Energetic (3)<br>Vital (3) |
| | | | Routine medical examinations | Performed | +++ (3) | Comfort (0) |
| | **Welfare compromise** | **Welfare Status** | General health | Sub-optimal | C (3) | Weakness (2)<br>Apathy (2)<br>Pain (2)<br>Discomfort (3) |

| | | | | | | |
|---|---|---|---|---|---|---|
| | | | | | | Gastrointestinal discomfort (0) |
| | | | | Poor health | D (3) | Debilitation (3)<br>Breathlessness (1) |
| | | | Reproductive state | Pregnancy | C (1) | Fatigue (2)<br>Discomfort (1)<br>Malaise (0) |
| | | | | Nursing | C (1) | Fatigue (0)<br>Discomfort (0) |
| | | | Tooth rakes | Presence | B (1) | Unease (2)<br>Pain (1) |
| | | | Eye conditions | Presence | D (3) | Discomfort (2)<br>Itchy (1)<br>Pain (1) |
| | | | Skin conditions | <35% skin marks | C (2) | Discomfort (3)<br>Unease (2)<br>Itchy (2) |
| | | | | > 35% skin marks | D (2) | Pain (2)<br>Malaise (1) |
| | | | Fecal/Blowhole Cortisol/DHEA ratio | ratio > 0 | D (2) | Discomfort (2)<br>Weakness (1)<br>Unease to breath (0)<br>Gastrointestinal discomfort (0) |
| | | | Fecal/Blowhole IgA levels | Below minimum requirements | D (2) | |
| | | **Welfare Alerting** | Water quality | Bad | D (2) | Discomfort (1) |
| | | | Pool cleaning | Absence | C (2) | Discomfort (1) |
| | | | Hygiene of fish preparation room | Bad | D (3) | Discomfort (2)<br>Gastrointestinal discomfort(2) |
| | | | Human-made-noises disturbance | Presence | B (3) | Acoustic discomfort (2)<br>Disorientation (1) |
| | | | Exposure to sunlight | Presence negative | D (2) | Discomfort (2)<br>Pain (2)<br>Malaise (1) |

| | | | | | | |
|---|---|---|---|---|---|---|
| | | | | Absence | C (1) | Discomfort (1) |
| | | | Frequency of feeding | Sub-optimal | C (1) | Weakness (1) |
| | | | Fish quality | Poor quality | D (3) | Gastrointestinal discomfort (2)<br>Weakness (0)<br>Nausea (0) |
| | | | Body condition scoring | Skinny | D (3) | Weakness (3)<br>Apathy (1)<br>Debilitated (2)<br>Malaise (1) |
| | | | | Overweight | B (3) | Unease to swim (0) |
| | | | Fish intake | Sub-optimal | D (3) | Weakness (2)<br>Discomfort (1) |
| | | | Suckling | Absence | D (3) | Weakness (2)<br>Debilitated (1) |
| | | | Rubbing behavior | Presence negative | C (1) | Itchy (2)<br>Uncomfortable (2) |
| | | | Latency to come to the trainer | (Over 1 minute) | C (2) | Discomfort (0)<br>Weakness (0)<br>Malaise (1)<br>Sickness (1) |
| | | | Breaking the trainer's interaction | (more than 3 times) | D (2) | Discomfort (0)<br>Weakness (0)<br>Malaise (1)<br>Sickness (1) |
| | | | Response to the trainer | no approach | D (2) | Weakness (0)<br>Malaise (1)<br>Sickness (1) |
| | | | | Approach-low motivation | C (2) | Discomfort (0) |
| | | | Routine medical examinations | Not performed | D (1) | Discomfort (0) |

| Domain | Valence | Category | Parameter | Conditions | Intensity Level/ confidence scores | Mental state and confidence scores |
|---|---|---|---|---|---|---|
| **D4** | **Welfare Enhancement** | **Welfare Status** | Solitary swimming | Slow positive | + (2) | Relaxed (2) |
| | | | Group swimming | Presence slow swim | ++ (2) | Safe (1)<br>Comfort (1) |
| | | | Swimming patterns | Slow swim | ++ (2) | Social support (2)<br>Relaxed (2)<br>Comfort (1) |
| | | | Play behaviors | Presence | +++ (3) | Fulfilled (2)<br>Playful (3)<br>Relaxed (3)<br>Comfortable (2)<br>Satisfied (2)<br>Pleasure (3) |
| | | | Socio-sexual behaviors | Presence normal rate | + (1) | Sexual fulfillment (3)<br>Socially supported (2)<br>Comfort (3)<br>Satisfied (3)<br>Aroused (3)<br>Pleasure (3) |
| | | | Aerial behaviors | Presence Positive | + (1) | Playfulness (1)<br>Content (0)<br>Vitality (3) |
| | | | Calf swim (echelon or infant swim) | Presence slow speed | ++ (2) | Social support (3)<br>Comfort (3)<br>Relaxed (1)<br>Safe (2) |
| | | | Rubbing behavior | Presence positive | + (1) | Comfort (2)<br>Pleasure (2) |
| | | | Exploratory behaviors | Presence | ++ (2) | Curious (3)<br>Satisfied (2)<br>In control (2)<br>Stimulated (3) |

| | | | | | | |
|---|---|---|---|---|---|---|
| | | | Floating at the surface | Presence positive | + (1) | Relaxed (1)<br>Comfort (1) |
| | | | Behavioral diversity index | Positive | +++ (2) | In control (2)<br>Curious (0)<br>Fulfilled (1)<br>Playful (1) |
| | | | Latency to come to the trainer | (0-1 minute) | ++ (2) | Engaged (3)<br>Interactive (3)<br>Motivated (3) |
| | | | Breaking the interaction with the trainer | (0-3 times) | ++ (2) | Engaged (3)<br>Interactive (3)<br>Motivated (3) |
| | | | Response to the trainer | Approach-good motivation | ++ (2) | Engaged (3)<br>Interactive (3)<br>Motivated (3) |
| | | | Anticipatory behaviors | Presence positive | + (2) | Excited (2)<br>Curious (1)<br>Anticipation (3) |
| | | | Interaction with enrichment devices | Presence | +++ (3) | Stimulated (3)<br>Curious (2)<br>Interactive (1)<br>Engaged (1)<br>Playful (1)<br>Sense of agency (2) |
| | | | Suckling | Presence | ++ (2) | Social (3)<br>Interactive (2)<br>Satisfied (1) |
| | | **Welfare Alerting** | Trainer's experience | Experienced | + (2) | Engaged(3)<br>Playful (1)<br>Cooperative (2) |
| | | | Unfamiliar human presence | Presence positive | + (1) | Curious (2)<br>Interactive (2)<br>Playful (2) |

| | | | | | | |
|---|---|---|---|---|---|---|
| | | | Pool size and depth | Optimal | + (2) | Comfort (1) |
| | | | Exposure to sunlight | Presence positive | +++ (2) | Pleasure (0)<br>Active (1) |
| | | | Opportunity of choice | Presence | +++ (3) | In control (3)<br>Stimulated (3)<br>Satisfied (2) |
| | | | Diversity of training sessions | Presence | +++ (2) | Stimulated (2)<br>Curious (0)<br>Engaged (1)<br>Playful (0) |
| | | | Social composition | Group housing positive | +++ (3) | Social support (3)<br>Affectionate (2)<br>Playful (2)<br>Sexually fulfilled (1)<br>Comfort (1) |
| | | | | Same sex grouping - Females | + (1) | Social support (2)<br>Affectionate (2)<br>Playful (1) |
| | | | Environmental enrichment | Presence | +++ (3) | Stimulated (3)<br>Curious (2)<br>Interactive (1)<br>Engaged (1)<br>Playful (1)<br>Sense of agency (2) |
| | | | Reproductive state | Pregnancy | ++ (2) | Fulfillment of reproduction drive (2) |
| | | | | Nursing | ++ (2) | Maternal affection (2) |
| | | | Frequency of feeding | Optimal | ++ (2) | Feeling in control (1)<br>Vitality (1)<br>Interactive (2) |
| | **Welfare Compromise** | **Welfare Status** | Route tracing | Presence | C (1) | Bored (2)<br>Frustrated (2)<br>Lack of agency (3) |

| | | | | | | |
|---|---|---|---|---|---|---|
| | | | | | | Distressed (1) |
| | | | Directional Swimming | Clock-wise/Counter-clockwise | C (1) | Bored (2)<br>Frustrated (2)<br>Lack of agency (3)<br>Distressed (1) |
| | | | Solitary swimming | Presence slow speed negative | C (3) | Loneliness (2)<br>Withdrawn (2) |
| | | | | Presence fast speed | B (3) | Vigilant (2)<br>Distressed (1)<br>Fear (1) |
| | | | Group swimming | Presence fast speed | D (3) | Distress (2)<br>Fear (2)<br>Annoyance (2) |
| | | | Swimming patterns | Fast swim | D (3) | Hyper vigilant (3)<br>Distressed (3)<br>Fear (2)<br>Annoyance (1) |
| | | | Aggressive behaviors: | Presence actor | C (2) | Fear (1)<br>Aroused (2)<br>Threatened (1)<br>Anxious (0)<br>Intolerant (2)<br>Angry (2)<br>Distressed (1) |
| | | | | Presence receiver | C (2) | Fear (2)<br>Aroused (2)<br>Threatened (2)<br>Anxious (0)<br>Angry (1)<br>Distressed (3) |
| | | | Play behaviors | Absence | D (1) | Bored (2)<br>Depressed (1)<br>Distressed (2) |

| | | | | | | |
|---|---|---|---|---|---|---|
| | | | Socio-sexual behaviors | Presence high rate | C (1) | Distressed (1)<br>Frustrated (1)<br>Anxious (2)<br>Bored (2)<br>Aroused (3) |
| | | | Aerial behaviors | Presence negative | D (2) | Fear (2)<br>Panic (2)<br>Hyper vigilant (2) |
| | | | Echelon/infant swim | Presence high speed | C (2) | Fearful (2)<br>Vigilant (2) |
| | | | | Absence mother | D (3) | Distressed (3)<br>Irritability (3)<br>Intolerance (3) |
| | | | | Absence calf | D (3) | Frustrated (1)<br>Insecure (2)<br>Fear (1)<br>Lack of maternal support (3) |
| | | | Rubbing behavior | Presence negative | C (1) | Frustration (0)<br>Distressed (0)<br>Boredom (0) |
| | | | Floating at the surface | Presence negative | C (1) | Bored (1)<br>Depressed (1) |
| | | | Behavioral diversity index | Negative | D (2) | Bored (2)<br>Frustrated (2)<br>Lack of control (1)<br>Distressed (1) |
| | | | Fleeing behaviors | Presence | C (3) | Fear (3)<br>Panic (2)<br>Frustrated (1)<br>Distressed (3)<br>Hyper vigilant (2) |
| | | | Latency to come to the trainers | (over 1 minute) | C (2) | Unmotivated (2)<br>Bored (2) |

| | | | | | | |
|---|---|---|---|---|---|---|
| | | | | | | Frustrated (2) |
| | | | Breaking the interaction with the trainer | (more than 3 times) | C (2) | Unmotivated (2)<br>Bored (2)<br>Frustrated (2) |
| | | | Response to the trainer | no approach | D (2) | Unmotivated (2) |
| | | | | Approach-low motivation | C (2) | Bored (2)<br>Frustrated (2) |
| | | | Anticipatory behaviors | Presence negative | C (1) | Hyper vigilant (3)<br>Bored (3) |
| | | | | Absence | B (2) | Unmotivated (2) |
| | | | Interaction with enrichment devices | Absence | D (3) | Frustration (1)<br>Bored (2) |
| | | | Suckling | Absence | D (3) | Depressed (1)<br>Frustrated (1) |
| | | **Welfare Alerting** | Pool cleaning | Presence | D (3) | Fear (3)<br>Uncomfortable (3)<br>Distressed (3)<br>Vigilance (3)<br>Insecurity (1)<br>Panic (1) |
| | | | Moving to a different pool | Presence | D (3) | Lost control (3)<br>Panic (3)<br>Vigilant (2)<br>Fear (3)<br>Feeling lack of social support (2)<br>Distressed (3) |
| | | | Trainer's Experience | Not-experienced | B (2) | Frustrated (2)<br>Bored (2)<br>Loss of control (1) |
| | | | Human-made-noises disturbances | Presence | D (3) | Agitated (2)<br>Annoyed (1)<br>Vigilant (3) |

| | | | | | | |
|---|---|---|---|---|---|---|
| | | | | | | Discomfort (1)<br>Loss of control (2)<br>Fear (1) |
| | | | Unfamiliar human presence | Presence negative | B (1) | Fear (1)<br>Anxiety (0)<br>Annoyance (2)<br>Hyper vigilance (2)<br>Loss of control (1) |
| | | | Pool size and depth | Sub-optimal | D (2) | Discomfort (3)<br>Boredom (3)<br>Frustration (2) |
| | | | Exposure to sunlight | Presence negative | D (2) | Annoyed (1)<br>Frustration (1) |
| | | | | Absence | D (1) | Depressed (1) |
| | | | Opportunity of choice | Absence | C (3) | Frustration (2)<br>Bored (2) |
| | | | Diversity of training sessions | Absence | D (2) | Bored (3) |
| | | | Social grouping | Presence negative | D (3) | Discomfort (2)<br>Withdrawn (2)<br>Fear (2)<br>Frustration (2)<br>Anxiety (2) |
| | | | Solitary housing | Presence | D (3) | Lonely (3)<br>Apathetic (1)<br>Frustrated (1)<br>Depressed (2)<br>Bored (2) |
| | | | Same sex-grouping | Males | B (1) | Sexually frustrated (2) |
| | | | | Females | B (1) | Sexually frustrated (0) |
| | | | Environmental enrichment | Absence | D (3) | Frustration (1)<br>Bored (2) |
| | | | Removal of food due to non-cooperation | Presence | C (3) | Frustration (3)<br>Loss of control (2) |

| | | | | | | |
|---|---|---|---|---|---|---|
| | | | Reproductive state | Pregnancy | B (1) | Irritability (0) |
| | | | | Nursing | B (1) | Irritability (0) |
| | | | Eye conditions | Presence | D (3) | Withdrawn (1)<br>Apathy (1) |
| | | | Skin conditions | <35% skin marks | C (2) | Fastidious (0)<br>Nervous (0) |
| | | | | > 35% skin marks | D (2) | Withdrawn (0) |
| | | | Fecal/Blowhole cortisol:DHEA ratio | Positive | D (2) | Distressed (2)<br>Anxious (2) |
| | | | Fecal/Blowhole IgA levels | below normal requirements | D (2) | Withdrawn (0)<br>Unease (0) |
| | | | Frequency of feedings | Sub-optimal | C (1) | Feeling lack of control (2)<br>Frustration (2)<br>Anxious (1) |
